# Supplementary material for: Sleep spindle maturity promotes slow oscillation-spindle coupling across child and adolescent development
Source: eLife. 2023 Nov 24;12:e83565. doi: 10.7554/eLife.83565 (PMC10672804; doi:10.7554/eLife.83565)
Supplement: Supplementary file 1. — (a) Type III analysis of variance table from a linear mixed-effects model on the effects of age group and sleep spindle topography on sleep spindle spectral peak frequency. (b) (A) Post-hoc comparisons for the topography effect on sleep spindle peak frequency based on estimated marginal means (B) Post-hoc comparisons for the age group effect on sleep spindle peak frequency based on estimated marginal means. (c) Type III analysis of variance table from a linear mixed-effects model on the effects of age group and sleep spindle topography on sleep spindle frequency. (d) Post-hoc comparisons for the sleep spindle frequency interaction effect based on estimated marginal means. (e) Type III analysis of variance table from a linear mixed-effects model on the effects of age group and sleep spindle topography on sleep spindle density. (f) Post-hoc comparisons for the sleep spindle density interaction effect based on estimated marginal means. (g) Type III analysis of variance table from a linear mixed-effects model on the effects of age group and sleep spindle topography on sleep spindle amplitude. (h) Post-hoc comparisons for the sleep spindle amplitude interaction effect based on estimated marginal means. (i) Type III analysis of variance table from a linear mixed-effects model on the effects of age group and sleep spindle type (development-specific and adult-like fast sleep spindles) on frequency. (j) Post-hoc comparisons for development-specific and adult-like fast sleep spindle frequency values within the four age groups (interaction effect) based on estimated marginal means. (k) Type III analysis of variance table from a linear mixed-effects model on the effects of age group and sleep spindle type (development-specific and adult-like fast sleep spindles) on density. (l) Post-hoc comparisons for development-specific and adult-like fast sleep spindle density within the four age groups (interaction effect) based on estimated marginal means. (m) Type III analysis of varianc [file elife-83565-supp1.docx]

**Supplementary file 1**

**Sleep spindle maturity promotes slow oscillation-spindle coupling
across child and adolescent development**

Ann-Kathrin Joechner^1^*, Michael A. Hahn^2,3,4^, Georg Gruber^5,6^, Kerstin Hoedlmoser^2,3^, and
Markus Werkle-Bergner^1^*

^1^ Center for Lifespan Psychology, Max Planck Institute for Human Development, Berlin, Germany

^2^ Department of Psychology, Laboratory for Sleep, Cognition and Consciousness Research, University of Salzburg, Salzburg, Austria

^3^ Centre for Cognitive Neuroscience Salzburg (CCNS), University of Salzburg, Salzburg, Austria

^4^ Hertie-Institute for Clinical Brain Research, University Medical Center Tuebingen, Tuebingen, Germany

^5^ Department of Psychiatry and Psychotherapy, Medical University of Vienna, Vienna, Austria

^6^ The Siesta Group, Vienna, Austria

* Corresponding authors: Ann-Kathrin Joechner (joechner@mpib-berlin.mpg.de) and
Markus Werkle-Bergner (werkle@mpib-berlin.mpg.de)

Table of contents

[Supplementary tables 1](#_Toc141792286)

[Individually identified sleep spindles 1](#_Toc141792287)

[Supplementary file 1a 1](#_Toc141792288)

[Supplementary file 1b 1](#_Toc141792289)

[Supplementary file 1c 2](#_Toc141792290)

[Supplementary file 1d 2](#_Toc141792291)

[Supplementary file 1e 3](#_Toc141792292)

[Supplementary file 1f 4](#_Toc141792293)

[Supplementary file 1g 5](#_Toc141792294)

[Supplementary file 1h 5](#_Toc141792295)

[Individually identified and adult-like fast sleep spindles 7](#_Toc141792296)

[Supplementary file 1i 7](#_Toc141792297)

[Supplementary file 1j 7](#_Toc141792298)

[Supplementary file 1k 9](#_Toc141792299)

[Supplementary file 1l 9](#_Toc141792300)

[Supplementary file 1m 11](#_Toc141792301)

[Supplementary file 1n 11](#_Toc141792302)

[Slow oscillations 13](#_Toc141792303)

[Supplementary file 1o 13](#_Toc141792304)

[Supplementary file 1p 13](#_Toc141792305)

[Supplementary file 1q 16](#_Toc141792306)

[Supplementary file 1r 16](#_Toc141792307)

[Supplementary file 1s 17](#_Toc141792308)

[Supplementary file 1t 17](#_Toc141792309)

[Association between sleep spindle and slow oscillation maturity with modulation strength 20](#_Toc141792310)

[Supplementary file 1u 20](#_Toc141792311)

[Supplementary file 1v 20](#_Toc141792312)

[Sleep architecture 21](#_Toc141792313)

[Supplementary file 1w 21](#_Toc141792314)

[Co-occurrence of sleep spindles and slow oscillations and vice versa 22](#_Toc141792315)

[Supplementary file 1x 22](#_Toc141792316)

[Supplementary file 1y 22](#_Toc141792317)

[Supplementary file 1z 22](#_Toc141792318)

[Supplementary file 1aa 23](#_Toc141792319)

[Supplementary file 1ab 23](#_Toc141792320)

[Supplementary file 1ac 23](#_Toc141792321)

# Supplementary tables

# Individually identified sleep spindles

### Supplementary file 1a

*Type III analysis of variance table from a linear mixed-effects model on the effects of age group and sleep spindle topography on sleep spindle spectral peak frequency*

| Predictor | Sum of squares | Mean square | *df*_Num_ | *df*_Den_ | *F* | *p* |
| --- | --- | --- | --- | --- | --- | --- |
| Age group | 23.79 | 7.93 | 3 | 89.40 | 50.77 | < .001 |
| Topography | 14.29 | 14.29 | 1 | 135.40 | 91.47 | < .001 |
| Age group*topography | 1.15 | 0.39 | 3 | 135.40 | 2.47 | .070 |

*Note*. *df*_Num_ indicates the degrees of freedom numerator. *df*_Den_ indicates the degrees of freedom denominator. Degrees of freedom were determined using Satterthwaite’s method.

### Supplementary file 1b

*(A) Post-hoc comparisons for the topography effect on sleep spindle peak frequency based on estimated marginal means (B) Post-hoc comparisons for the age group effect on sleep spindle peak frequency based on estimated marginal means*

| **A** |  |  |  |  |  |
| --- | --- | --- | --- | --- | --- |
| Contrast | Estimate | *SE* | *df* | *t* | *p*_adj_ |
| Frontal – Centro-parietal | -0.53 | 0.06 | 135 | -9.56 | < .001 |

| **B** |  | |  |  |  |  |
| --- | --- | --- | --- | --- | --- | --- |
| Contrast | | Estimate | *SE* | *df* | *t* | *p_adj_* |
| 5–6-year-olds – 8–11-year-olds | | -0.46 | 0.13 | 84.4 | -3.54 | .004 |
| 5–6-year-olds – 14–18-year-olds | | -1.06 | 0.13 | 84.4 | -8.17 | < .001 |
| 5–6-year-olds – 20–26-year-olds | | -1.28 | 0.15 | 84.4 | -8.51 | < .001 |
| 8–11-year-olds – 14–18-year-olds | | -0.60 | 0.07 | 135.4 | -8.72 | < .001 |
| 8–11-year-olds – 20–26-year-olds | | -0.83 | 0.14 | 84.4 | -5.82 | < .001 |
| 14–18-year-olds – 20–26-year-olds | | -0.22 | 0.14 | 84.4 | -1.58 | .711 |

*Note*. Degrees of freedom were determined using Satterthwaite’s method. *P_adj_*-values are Bonferroni corrected. *SE*= standard error, *df*= degrees of freedom.

### Supplementary file 1c

*Type III analysis of variance table from a linear mixed-effects model on the effects of age group and sleep spindle topography on sleep spindle frequency*

| Predictor | Sum of squares | Mean square | *df*_Num_ | *df*_Den_ | *F* | *p* |
| --- | --- | --- | --- | --- | --- | --- |
| Age group | 23.59 | 7.86 | 3 | 86.74 | 69.80 | < .001 |
| Topography | 19.55 | 19.55 | 1 | 134.73 | 173.55 | < .001 |
| Age group*topography | 1.37 | 0.46 | 3 | 134.73 | 4.04 | .009 |

*Note*. *df*_Num_ indicates the degrees of freedom numerator. *df*_Den_ indicates the degrees of freedom denominator. Degrees of freedom were determined using Satterthwaite’s method.

### Supplementary file 1d

*Post-hoc comparisons for the sleep spindle frequency interaction effect based on estimated marginal means*

| Contrast | Estimate | *SE* | *df* | *t* | *p*_adj_ |
| --- | --- | --- | --- | --- | --- |
| 5–6-y F – 8–11-y F | -0.56 | 0.15 | 112.39 | -3.69 | **.010** |
| 5–6-y F – 14–18-y F | -1.17 | 0.15 | 112.39 | -7.74 | **< .001** |
| 5–6-y F – 20–26-y F | -1.15 | 0.18 | 112.39 | -6.57 | **< .001** |
| 5–6-y F - 5–6-y CP | -0.54 | 0.10 | 134.73 | -5.62 | **< .001** |
| 5–6-y F - 8–11-y CP | -1.00 | 0.15 | 112.39 | -6.65 | **< .001** |
| 5–6-y F - 14–18-y CP | -1.74 | 0.15 | 112.39 | -11.54 | **< .001** |
| 5–6-y F - 20–26-y CP | -2.07 | 0.18 | 112.39 | -11.83 | **< .001** |
| 8–11-y F - 14–18-y F | -0.61 | 0.08 | 134.73 | -7.37 | **< .001** |
| 8–11-y F - 20–26-y F | -0.59 | 0.16 | 112.39 | -3.61 | **.013** |
| 8–11-y F - 5–6-y CP | 0.01 | 0.15 | 112.39 | 0.08 | 1.000 |
| 8–11-y F - 8–11-y CP | -0.44 | 0.08 | 134.73 | -5.38 | **< .001** |
| 8–11-y F - 14–18-y CP | -1.18 | 0.08 | 134.73 | -14.31 | **< .001** |
| 8–11-y F - 20–26-y CP | -1.52 | 0.16 | 112.39 | -9.21 | **< .001** |
| 14–18-y F - 20–26-y F | 0.02 | 0.16 | 112.39 | 0.09 | 1.000 |
| 14–18-y F - 5–6-y CP | 0.62 | 0.15 | 112.39 | 4.12 | **.002** |
| 14–18-y F - 8–11-y CP | 0.16 | 0.08 | 134.73 | 1.99 | 1.000 |
| 14–18-y F - 14–18-y CP | -0.57 | 0.08 | 134.73 | -6.94 | **< .001** |
| 14–18-y F - 20–26-y CP | -0.91 | 0.16 | 112.39 | -5.51 | **< .001** |
| 20–26-y F - 5–6-y CP | 0.61 | 0.18 | 112.39 | 3.46 | **.022** |
| 20–26-y F - 8–11-y CP | 0.15 | 0.16 | 112.39 | 0.91 | 1.000 |
| 20–26-y F - 14–18-y CP | -0.59 | 0.16 | 112.39 | -3.58 | **.015** |
| 20–26-y F - 20–26-y CP | -0.92 | 0.11 | 134.73 | -8.24 | **< .001** |
| 5–6-y CP - 8–11-y CP | -0.46 | 0.15 | 112.39 | -3.03 | .084 |
| 5–6-y CP - 14–18-y CP | -1.19 | 0.15 | 112.39 | -7.93 | **< .001** |
| 5–6-y CP - 20–26-y CP | -1.53 | 0.18 | 112.39 | -8.72 | **< .001** |
| 8–11-y CP - 14–18-y CP | -0.74 | 0.08 | 134.73 | -8.92 | **< .001** |
| 8–11-y CP - 20–26-y CP | -1.07 | 0.16 | 112.39 | -6.51 | **< .001** |
| 14–18-y CP - 20–26-y CP | -0.33 | 0.16 | 112.39 | -2.03 | 1.000 |

*Note*. Degrees of freedom were determined using Satterthwaite’s method. *P_adj_*-values are Bonferroni corrected. *SE*= standard error, *df*= degrees of freedom.

### Supplementary file 1e

*Type III analysis of variance table from a linear mixed-effects model on the effects of age group and sleep spindle topography on sleep spindle density*

| Predictor | Sum of squares | Mean square | *df*_Num_ | *df*_Den_ | *F* | *p* |
| --- | --- | --- | --- | --- | --- | --- |
| Age group | 1.44 | 0.48 | 3 | 88.71 | 10.29 | < .001 |
| Topography | 0.78 | 0.78 | 1 | 136.76 | 16.70 | < .001 |
| Age group*topography | 1.65 | 0.55 | 3 | 136.76 | 11.73 | < .001 |

*Note*. *df*_Num_ indicates the degrees of freedom numerator. *df*_Den_ indicates the degrees of freedom denominator. Degrees of freedom were determined using Satterthwaite’s method.

### Supplementary file 1f

*Post-hoc comparisons for the sleep spindle density interaction effect based on estimated marginal means*

| Contrast | Estimate | *SE* | *df* | *t* | *p*_adj_ |
| --- | --- | --- | --- | --- | --- |
| 5–6-y F – 8–11-y F | -0.43 | 0.10 | 111.71 | -4.26 | **.001** |
| 5–6-y F – 14–18-y F | -0.22 | 0.10 | 111.71 | -2.21 | .825 |
| 5–6-y F – 20–26-y F | 0.02 | 0.12 | 111.71 | 0.19 | 1.000 |
| 5–6-y F - 5–6-y CP | 0.36 | 0.06 | 136.76 | 5.69 | **< .001** |
| 5–6-y F - 8–11-y CP | -0.18 | 0.10 | 111.71 | -1.80 | 1.000 |
| 5–6-y F - 14–18-y CP | -0.18 | 0.10 | 111.71 | -1.76 | 1.000 |
| 5–6-y F - 20–26-y CP | -0.13 | 0.12 | 111.71 | -1.09 | 1.000 |
| 8–11-y F - 14–18-y F | 0.21 | 0.05 | 136.76 | 3.86 | **.005** |
| 8–11-y F - 20–26-y F | 0.45 | 0.11 | 111.71 | 4.11 | **.002** |
| 8–11-y F - 5–6-y CP | 0.78 | 0.10 | 111.71 | 7.82 | **< .001** |
| 8–11-y F - 8–11-y CP | 0.25 | 0.05 | 136.76 | 4.62 | **< .001** |
| 8–11-y F - 14–18-y CP | 0.25 | 0.05 | 136.76 | 4.68 | **< .001** |
| 8–11-y F - 20–26-y CP | 0.30 | 0.11 | 111.71 | 2.74 | .200 |
| 14–18-y F - 20–26-y F | 0.24 | 0.11 | 111.71 | 2.22 | .790 |
| 14–18-y F - 5–6-y CP | 0.58 | 0.10 | 111.71 | 5.76 | **< .001** |
| 14–18-y F - 8–11-y CP | 0.04 | 0.05 | 136.76 | 0.77 | 1.000 |
| 14–18-y F - 14–18-y CP | 0.04 | 0.05 | 136.76 | 0.83 | 1.000 |
| 14–18-y F - 20–26-y CP | 0.09 | 0.11 | 111.71 | 0.86 | 1.000 |
| 20–26-y F - 5–6-y CP | 0.33 | 0.12 | 111.71 | 2.87 | .138 |
| 20–26-y F - 8–11-y CP | -0.20 | 0.11 | 111.71 | -1.85 | 1.000 |
| 20–26-y F - 14–18-y CP | -0.20 | 0.11 | 111.71 | -1.82 | 1.000 |
| 20–26-y F - 20–26-y CP | -0.15 | 0.07 | 136.76 | -2.07 | 1.000 |
| 5–6-y CP - 8–11-y CP | -0.53 | 0.10 | 111.71 | -5.35 | **< .001** |
| 5–6-y CP - 14–18-y CP | -0.53 | 0.10 | 111.71 | -5.32 | **< .001** |
| 5–6-y CP - 20–26-y CP | -0.48 | 0.12 | 111.71 | -4.15 | **.002** |
| 8–11-y CP - 14–18-y CP | 0.00 | 0.05 | 136.76 | 0.06 | 1.000 |
| 8–11-y CP - 20–26-y CP | 0.05 | 0.11 | 111.71 | 0.48 | 1.000 |
| 14–18-y CP - 20–26-y CP | 0.05 | 0.11 | 111.71 | 0.45 | 1.000 |

*Note*. Degrees of freedom were determined using Satterthwaite’s method. *P_adj_*-values are Bonferroni corrected. *SE*= standard error, *df*= degrees of freedom. F=frontal, CP= centro-parietal.

### Supplementary file 1g

*Type III analysis of variance table from a linear mixed-effects model on the effects of age group and sleep spindle topography on sleep spindle amplitude*

| Predictor | Sum of squares | Mean square | *df*_Num_ | *df*_Den_ | *F* | *p* |
| --- | --- | --- | --- | --- | --- | --- |
| Age group | 4,354.00 | 1,451.33 | 3 | 94.31 | 44.53 | < .001 |
| Topography | 7,836.03 | 7,836.03 | 1 | 140.21 | 240.43 | < .001 |
| Age group*topography | 532.85 | 177.62 | 3 | 140.21 | 5.45 | .001 |

*Note*. *df*_Num_ indicates the degrees of freedom numerator. *df*_Den_ indicates the degrees of freedom denominator. Degrees of freedom were determined using Satterthwaite’s method.

### Supplementary file 1h

*Post-hoc comparisons for the sleep spindle amplitude interaction effect based on estimated marginal means*

| Contrast | Estimate | *SE* | *df* | *t* | *p*_adj_ |
| --- | --- | --- | --- | --- | --- |
| 5–6-y F – 8–11-y F | -8.18 | 2.22 | 137.00 | -3.68 | **.009** |
| 5–6-y F – 14–18-y F | 5.04 | 2.22 | 137.00 | 2.27 | .698 |
| 5–6-y F – 20–26-y F | 11.15 | 2.58 | 137.00 | 4.32 | .**001** |
| 5–6-y F - 5–6-y CP | 11.54 | 1.65 | 140.21 | 7.01 | **< .001** |
| 5–6-y F - 8–11-y CP | 9.33 | 2.22 | 137.00 | 4.20 | **.001** |
| 5–6-y F - 14–18-y CP | 16.70 | 2.22 | 137.00 | 7.52 | **< .001** |
| 5–6-y F - 20–26-y CP | 20.17 | 2.58 | 137.00 | 7.81 | **< .001** |
| 8–11-y F - 14–18-y F | 13.21 | 1.41 | 140.21 | 9.40 | **< .001** |
| 8–11-y F - 20–26-y F | 19.33 | 2.43 | 137.00 | 7.97 | **< .001** |
| 8–11-y F - 5–6-y CP | 19.72 | 2.22 | 137.00 | 8.88 | **< .001** |
| 8–11-y F - 8–11-y CP | 17.51 | 1.41 | 140.21 | 12.46 | **< .001** |
| 8–11-y F - 14–18-y CP | 24.87 | 1.41 | 140.21 | 17.70 | **< .001** |
| 8–11-y F - 20–26-y CP | 28.35 | 2.43 | 137.00 | 11.69 | **< .001** |
| 14–18-y F - 20–26-y F | 6.12 | 2.43 | 137.00 | 2.52 | .360 |
| 14–18-y F - 5–6-y CP | 6.51 | 2.22 | 137.00 | 2.93 | .111 |
| 14–18-y F - 8–11-y CP | 4.29 | 1.41 | 140.21 | 3.05 | .075 |
| 14–18-y F - 14–18-y CP | 11.66 | 1.41 | 140.21 | 8.30 | **< .001** |
| 14–18-y F - 20–26-y CP | 15.14 | 2.43 | 137.00 | 6.24 | **< .001** |
| 20–26-y F - 5–6-y CP | 0.39 | 2.58 | 137.00 | 0.15 | 1.000 |
| 20–26-y F - 8–11-y CP | -1.82 | 2.43 | 137.00 | -0.75 | 1.000 |
| 20–26-y F - 14–18-y CP | 5.54 | 2.43 | 137.00 | 2.28 | .668 |
| 20–26-y F - 20–26-y CP | 9.02 | 1.90 | 140.21 | 4.74 | **< .001** |
| 5–6-y CP - 8–11-y CP | -2.21 | 2.22 | 137.00 | -1.00 | 1.000 |
| 5–6-y CP - 14–18-y CP | 5.15 | 2.22 | 137.00 | 2.32 | .613 |
| 5–6-y CP - 20–26-y CP | 8.63 | 2.58 | 137.00 | 3.34 | **.030** |
| 8–11-y CP - 14–18-y CP | 7.37 | 1.41 | 140.21 | 5.24 | **< .001** |
| 8–11-y CP - 20–26-y CP | 10.84 | 2.43 | 137.00 | 4.47 | **< .001** |
| 14–18-y CP - 20–26-y CP | 3.48 | 2.43 | 137.00 | 1.43 | 1.000 |

*Note*. Degrees of freedom were determined using Satterthwaite’s method. *P_adj_*-values are Bonferroni corrected. *SE*= standard error, *df*= degrees of freedom. F=frontal, CP= centro-parietal*.*

# Individually identified and adult-like fast sleep spindles

### Supplementary file 1i

*Type III analysis of variance table from a linear mixed-effects model on the effects of age group and sleep spindle type (development-specific and adult-like fast sleep spindles) on frequency*

| Predictor | Sum of squares | Mean square | *df*_Num_ | *df*_Den_ | *F* | *p* |
| --- | --- | --- | --- | --- | --- | --- |
| Age group | 13.64 | 4.55 | 3 | 90.45 | 55.11 | < .001 |
| Spindle type | 29.96 | 29.96 | 1 | 135.85 | 363.03 | < .001 |
| Age group*spindle type | 12.36 | 4.12 | 3 | 135.85 | 49.94 | < .001 |

*Note*. *df*_Num_ indicates the degrees of freedom numerator. *df*_Den_ indicates the degrees of freedom denominator. Degrees of freedom were determined using Satterthwaite’s method.

### Supplementary file 1j

*Post-hoc comparisons for development-specific and adult-like fast sleep spindle frequency values within the four age groups (interaction effect) based on estimated marginal means*

| Contrast | Estimate | *SE* | *df* | *t* | *p*_adj_ |
| --- | --- | --- | --- | --- | --- |
| 5–6-y adult-like –  8–11-y adult-like | 0.21 | 0.11 | 142.98 | 2.01 | 1.000 |
| 5–6-y adult-like –  14–18-y adult-like | -0.02 | 0.11 | 142.98 | -0.22 | 1.000 |
| 5–6-y adult-like –  20–26-y adult-like | -0.24 | 0.12 | 142.98 | -1.94 | 1.000 |
| 5–6-y adult-like –  5–6-y development-specific | 1.55 | 0.08 | 135.85 | 18.70 | **< .001** |
| 5–6-y adult-like –  8–11-y development-specific | 1.09 | 0.11 | 142.98 | 10.39 | **< .001** |
| 5–6-y adult-like –  14–18-y development-specific | 0.36 | 0.11 | 142.98 | 3.39 | **.025** |
| 5–6-y adult-like –  20–26-y development-specific | 0.02 | 0.12 | 142.98 | 0.19 | 1.000 |
| 8–11-y adult-like –  14–18-y adult-like | -0.23 | 0.07 | 135.85 | -3.32 | **.033** |
| 8–11-y adult-like –  20–26-y adult-like | -0.45 | 0.12 | 142.98 | -3.91 | **.004** |
| 8–11-y adult-like –  5–6-y development-specific | 1.34 | 0.11 | 142.98 | 12.71 | **< .001** |
| 8–11-y adult-like –  8–11-y development-specific | 0.88 | 0.07 | 135.85 | 12.48 | **< .001** |
| 8–11-y adult-like –  14–18-y development-specific | 0.15 | 0.07 | 135.85 | 2.06 | 1.000 |
| 8–11-y adult-like –  20–26-y development-specific | -0.19 | 0.12 | 142.98 | -1.63 | 1.000 |
| 14–18-y adult-like –  20–26-y adult-like | -0.22 | 0.12 | 142.98 | -1.87 | 1.000 |
| 14–18-y adult-like –  5–6-y development-specific | 1.57 | 0.11 | 142.98 | 14.94 | **< .001** |
| 14–18-y adult-like –  8–11-y development-specific | 1.12 | 0.07 | 135.85 | 15.80 | **< .001** |
| 14–18-y adult-like –  14–18-y development-specific | 0.38 | 0.07 | 135.85 | 5.37 | **< .001** |
| 14–18-y adult-like –  20–26-y development-specific | 0.05 | 0.12 | 142.98 | 0.40 | 1.000 |
| 20–26-y adult-like –  5–6-y development-specific | 1.79 | 0.12 | 142.98 | 14.61 | **< .001** |
| 20–26-y adult-like –  8–11-y development-specific | 1.33 | 0.12 | 142.98 | 11.58 | **< .001** |
| 20–26-y adult-like –  14–18-y development-specific | 0.60 | 0.12 | 142.98 | 5.17 | **< .001** |
| 20–26-y adult-like –  20–26-y development-specific | 0.26 | 0.10 | 135.85 | 2.73 | .199 |
| 5–6-y development-specific –  8–11-y development-specific | -0.46 | 0.11 | 142.98 | -4.33 | **.001** |
| 5–6-y development-specific – 14–18-y development-specific | -1.19 | 0.11 | 142.98 | -11.33 | **< .001** |
| 5–6-y development-specific –  20–26-y development-specific | -1.53 | 0.12 | 142.98 | -12.47 | **< .001** |
| 8–11-y development-specific –  14–18-y development-specific | -0.74 | 0.07 | 135.85 | -10.42 | **< .001** |
| 8–11-y development-specific –  20–26-y development-specific | -1.07 | 0.12 | 142.98 | -9.30 | **< .001** |
| 14–18-y development-specific –  20–26-y development-specific | -0.33 | 0.12 | 142.98 | -2.90 | .122 |

*Note*. Degrees of freedom were determined using Satterthwaite’s method. *P_adj_*-values are Bonferroni corrected. *SE*= standard error, *df*= degrees of freedom.

### Supplementary file 1k

*Type III analysis of variance table from a linear mixed-effects model on the effects of age group and sleep spindle type (development-specific and adult-like fast sleep spindles) on density*

| Predictor | Sum of squares | Mean square | *df*_Num_ | *df*_Den_ | *F* | *p* |
| --- | --- | --- | --- | --- | --- | --- |
| Age group | 4.44 | 1.48 | 3 | 90.72 | 31.75 | < .001 |
| Spindle type | 3.71 | 3.71 | 1 | 138.60 | 79.51 | < .001 |
| Age group*spindle type | 2.70 | 0.90 | 3 | 138.60 | 19.28 | < .001 |

*Note*. *df*_Num_ indicates the degrees of freedom numerator. *df*_Den_ indicates the degrees of freedom denominator. Degrees of freedom were determined using Satterthwaite’s method.

### Supplementary file 1l

*Post-hoc comparisons for development-specific and adult-like fast sleep spindle density within the four age groups (interaction effect) based on estimated marginal means*

| Contrast | Estimate | *SE* | *df* | *t* | *p*_adj_ |
| --- | --- | --- | --- | --- | --- |
| 5–6-y adult-like –  8–11-y adult-like | -0.50 | 0.10 | 113.55 | -4.98 | **< .001** |
| 5–6-y adult-like –  14–18-y adult-like | -0.93 | 0.10 | 113.55 | -9.31 | **< .001** |
| 5–6-y adult-like –  20–26-y adult-like | -0.95 | 0.12 | 113.55 | -8.18 | **< .001** |
| 5–6-y adult-like –  5–6-y development-specific | -0.48 | 0.06 | 138.60 | -7.66 | **< .001** |
| 5–6-y adult-like –  8–11-y development-specific | -1.01 | 0.10 | 113.55 | -10.13 | **< .001** |
| 5–6-y adult-like –  14–18-y development-specific | -1.01 | 0.10 | 113.55 | -10.10 | **< .001** |
| 5–6-y adult-like –  20–26-y development-specific | -0.96 | 0.12 | 113.55 | -8.27 | **< .001** |
| 8–11-y adult-like –  14–18-y adult-like | -0.43 | 0.05 | 138.60 | -8.13 | **< .001** |
| 8–11-y adult-like –  20–26-y adult-like | -0.45 | 0.11 | 113.55 | -4.15 | **.002** |
| 8–11-y adult-like –  5–6-y development-specific | 0.02 | 0.10 | 113.55 | 0.20 | 1.000 |
| 8–11-y adult-like –  8–11-y development-specific | -0.51 | 0.05 | 138.60 | -9.69 | **< .001** |
| 8–11-y adult-like –  14–18-y development-specific | -0.51 | 0.05 | 138.60 | -9.63 | **< .001** |
| 8–11-y adult-like –  20–26-y development-specific | -0.46 | 0.11 | 113.55 | -4.24 | **.001** |
| 14–18-y adult-like –  20–26-y adult-like | -0.02 | 0.11 | 113.55 | -0.19 | 1.000 |
| 14–18-y adult-like –  5–6-y development-specific | 0.45 | 0.10 | 113.55 | 4.52 | **< .001** |
| 14–18-y adult-like –  8–11-y development-specific | -0.08 | 0.05 | 138.60 | -1.56 | 1.000 |
| 14–18-y adult-like –  14–18-y development-specific | -0.08 | 0.05 | 138.60 | -1.50 | 1.000 |
| 14–18-y adult-like –  20–26-y development-specific | -0.03 | 0.11 | 113.55 | -0.28 | 1.000 |
| 20–26-y adult-like –  5–6-y development-specific | 0.47 | 0.12 | 113.55 | 4.07 | **.002** |
| 20–26-y adult-like –  8–11-y development-specific | -0.06 | 0.11 | 113.55 | -0.57 | 1.000 |
| 20–26-y adult-like –  14–18-y development-specific | -0.06 | 0.11 | 113.55 | -0.54 | 1.000 |
| 20–26-y adult-like –  20–26-y development-specific | -0.01 | 0.07 | 138.60 | -0.13 | 1.000 |
| 5–6-y development-specific –  8–11-y development-specific | -0.53 | 0.10 | 113.55 | -5.35 | **< .001** |
| 5–6-y development-specific –  14–18-y development-specific | -0.53 | 0.10 | 113.55 | -5.32 | **< .001** |
| 5–6-y development-specific –  20–26-y development-specific | -0.48 | 0.12 | 113.55 | -4.15 | **.002** |
| 8–11-y development-specific –  14–18-y development-specific | 0.00 | 0.05 | 138.60 | 0.06 | 1.000 |
| 8–11-y development-specific –  20–26-y development-specific | 0.05 | 0.11 | 113.55 | 0.48 | 1.000 |
| 14–18-y development-specific –  20–26-y development-specific | 0.05 | 0.11 | 113.55 | 0.45 | 1.000 |

*Note*. Degrees of freedom were determined using Satterthwaite’s method. *P_adj_*-values are Bonferroni corrected. *SE*= standard error, *df*= degrees of freedom.

### Supplementary file 1m

*Type III analysis of variance table from a linear mixed-effects model on the effects of age group and sleep spindle type (development-specific and adult-like fast sleep spindles) on amplitude*

| Predictor | Sum of squares | Mean square | *df*_Num_ | *df*_Den_ | *F* | *p* |
| --- | --- | --- | --- | --- | --- | --- |
| Age group | 1,449.33 | 483.11 | 3 | 97.07 | 37.91 | < .001 |
| Spindle type | 262.35 | 262.35 | 1 | 142.56 | 20.59 | < .001 |
| Age group*spindle type | 187.45 | 62.48 | 3 | 142.56 | 4.90 | .003 |

*Note*. *df*_Num_ indicates the degrees of freedom numerator. *df*_Den_ indicates the degrees of freedom denominator. Degrees of freedom were determined using Satterthwaite’s method.

### Supplementary file 1n

*Post-hoc comparisons for development-specific and adult-like fast sleep spindle amplitude within the four age groups (interaction effect) based on estimated marginal means*

| Contrast | Estimate | *SE* | *df* | *t* | *p*_adj_ |
| --- | --- | --- | --- | --- | --- |
| 5–6-y adult-like –  8–11-y adult-like | -2.76 | 1.38 | 140.15 | -2.00 | 1.000 |
| 5–6-y adult-like –  14–18-y adult-like | 1.55 | 1.38 | 140.15 | 1.12 | 1.000 |
| 5–6-y adult-like –  20–26-y adult-like | 4.00 | 1.61 | 140.15 | 2.49 | .392 |
| 5–6-y adult-like –  5–6-y development-specific | -4.47 | 1.03 | 142.56 | -4.34 | **.001** |
| 5–6-y adult-like –  8–11-y development-specific | -6.68 | 1.38 | 140.15 | -4.83 | **< .001** |
| 5–6-y adult-like –  14–18-y development-specific | 0.68 | 1.38 | 140.15 | 0.49 | 1.000 |
| 5–6-y adult-like –  20–26-y development-specific | 4.16 | 1.61 | 140.15 | 2.59 | .298 |
| 8–11-y adult-like –  14–18-y adult-like | 4.31 | 0.88 | 142.56 | 4.90 | **< .001** |
| 8–11-y adult-like –  20–26-y adult-like | 6.76 | 1.51 | 140.15 | 4.48 | **< .001** |
| 8–11-y adult-like –  5–6-y development-specific | -1.71 | 1.38 | 140.15 | -1.24 | 1.000 |
| 8–11-y adult-like –  8–11-y development-specific | -3.92 | 0.88 | 142.56 | -4.47 | **< .001** |
| 8–11-y adult-like –  14–18-y development-specific | 3.44 | 0.88 | 142.56 | 3.92 | **.004** |
| 8–11-y adult-like –  20–26-y development-specific | 6.92 | 1.51 | 140.15 | 4.58 | **< .001** |
| 14–18-y adult-like –  20–26-y adult-like | 2.45 | 1.51 | 140.15 | 1.62 | 1.000 |
| 14–18-y adult-like –  5–6-y development-specific | -6.02 | 1.38 | 140.15 | -4.35 | **.001** |
| 14–18-y adult-like –  8–11-y development-specific | -8.23 | 0.88 | 142.56 | -9.37 | **< .001** |
| 14–18-y adult-like –  14–18-y development-specific | -0.87 | 0.88 | 142.56 | -0.99 | 1.000 |
| 14–18-y adult-like –  20–26-y development-specific | 2.61 | 1.51 | 140.15 | 1.73 | 1.000 |
| 20–26-y adult-like –  5–6-y development-specific | -8.47 | 1.61 | 140.15 | -5.27 | **< .001** |
| 20–26-y adult-like –  8–11-y development-specific | -10.68 | 1.51 | 140.15 | -7.07 | **< .001** |
| 20–26-y adult-like –  14–18-y development-specific | -3.32 | 1.51 | 140.15 | -2.20 | .830 |
| 20–26-y adult-like –  20–26-y development-specific | 0.16 | 1.19 | 142.56 | 0.14 | 1.000 |
| 5–6-y development-specific –  8–11-y development-specific | -2.21 | 1.38 | 140.15 | -1.60 | 1.000 |
| 5–6-y development-specific –  14–18-y development-specific | 5.15 | 1.38 | 140.15 | 3.73 | **.008** |
| 5–6-y development-specific –  20–26-y development-specific | 8.63 | 1.61 | 140.15 | 5.37 | **< .001** |
| 8–11-y development-specific –  14–18-y development-specific | 7.37 | 0.88 | 142.56 | 8.38 | **< .001** |
| 8–11-y development-specific –  20–26-y development-specific | 10.84 | 1.51 | 140.15 | 7.18 | **< .001** |
| 14–18-y development-specific –  20–26-y development-specific | 3.48 | 1.51 | 140.15 | 2.30 | .636 |

*Note*. Degrees of freedom were determined using Satterthwaite’s method. *P_adj_*-values are Bonferroni corrected. *SE*= standard error, *df*= degrees of freedom.

# Slow oscillations

### Supplementary file 1o

*Type III analysis of variance table from a linear mixed-effects model on the effects of age group and slow oscillation topography on slow oscillation frequency*

| Predictor | Sum of squares | Mean square | *df*_Num_ | *df*_Den_ | *F* | *p* |
| --- | --- | --- | --- | --- | --- | --- |
| Age group | 0.03 | 0.01 | 3 | 96.12 | 51.64 | < .001 |
| Topography | 0.03 | 0.02 | 2 | 238.15 | 81.04 | < .001 |
| Age group*topography | 0.02 | 0.00 | 6 | 238.15 | 13.10 | < .001 |

*Note*. *df*_Num_ indicates the degrees of freedom numerator. *df*_Den_ indicates the degrees of freedom denominator. Degrees of freedom were determined using Satterthwaite’s method.

### Supplementary file 1p

*Post-hoc comparisons for the slow oscillation frequency interaction effect based on estimated marginal means*

| Contrast | Estimate | *SE* | *df* | *t* | *p*_adj_ |
| --- | --- | --- | --- | --- | --- |
| 5–6-y F - 8–11-y F | -0.01 | 0.01 | 177.11 | -1.52 | 1.000 |
| 5–6-y F - 14–18-y F | 0.01 | 0.01 | 177.11 | 1.10 | 1.000 |
| 5–6-y F - 20–26-y F | 0.00 | 0.01 | 177.11 | 0.82 | 1.000 |
| 5–6-y F - 5–6-y CP | 0.00 | 0.00 | 238.15 | 0.15 | 1.000 |
| 5–6-y F - 8–11-y CP | -0.00 | 0.01 | 177.11 | -0.46 | 1.000 |
| 5–6-y F - 14–18-y CP | 0.01 | 0.01 | 177.11 | 2.72 | .481 |
| 5–6-y F - 20–26-y CP | 0.02 | 0.01 | 177.11 | 2.68 | .537 |
| 5–6-y F - 5–6-y O | 0.01 | 0.00 | 238.15 | 1.42 | 1.000 |
| 5–6-y F - 8–11-y O | 0.00 | 0.01 | 177.11 | 0.40 | 1.000 |
| 5–6-y F - 14–18-y O | 0.04 | 0.01 | 177.11 | 8.20 | **< .001** |
| 5–6-y F - 20–26-y O | 0.05 | 0.01 | 177.11 | 8.41 | **< .001** |
| 8–11-y F - 14–18-y F | 0.01 | 0.00 | 238.15 | 3.86 | **.010** |
| 8–11-y F - 20–26-y F | 0.01 | 0.01 | 177.11 | 2.26 | 1.000 |
| 8–11-y F - 5–6-y CP | 0.01 | 0.01 | 177.11 | 1.64 | 1.000 |
| 8–11-y F - 8–11-y CP | 0.01 | 0.00 | 238.15 | 1.57 | 1.000 |
| 8–11-y F - 14–18-y CP | 0.02 | 0.00 | 238.15 | 6.25 | **< .001** |
| 8–11-y F - 20–26-y CP | 0.02 | 0.01 | 177.11 | 4.24 | **.002** |
| 8–11-y F - 5–6-y O | 0.01 | 0.01 | 177.11 | 2.65 | .577 |
| 8–11-y F - 8–11-y O | 0.01 | 0.00 | 238.15 | 2.84 | .321 |
| 8–11-y F - 14–18-y O | 0.05 | 0.00 | 238.15 | 14.35 | **< .001** |
| 8–11-y F - 20–26-y O | 0.06 | 0.01 | 177.11 | 10.34 | **< .001** |
| 14–18-y F - 20–26-y F | -0.00 | 0.01 | 177.11 | -0.13 | 1.000 |
| 14–18-y F - 5–6-y CP | -0.01 | 0.01 | 177.11 | -0.98 | 1.000 |
| 14–18-y F - 8–11-y CP | -0.01 | 0.00 | 238.15 | -2.29 | 1.000 |
| 14–18-y F - 14–18-y CP | 0.01 | 0.00 | 238.15 | 2.39 | 1.000 |
| 14–18-y F - 20–26-y CP | 0.01 | 0.01 | 177.11 | 1.85 | 1.000 |
| 14–18-y F - 5–6-y O | 0.00 | 0.01 | 177.11 | 0.03 | 1.000 |
| 14–18-y F - 8–11-y O | -0.00 | 0.00 | 238.15 | -1.02 | 1.000 |
| 14–18-y F - 14–18-y O | 0.04 | 0.00 | 238.15 | 10.48 | **< .001** |
| 14–18-y F - 20–26-y O | 0.04 | 0.01 | 177.11 | 7.95 | **< .001** |
| 20–26-y F - 5–6-y CP | -0.00 | 0.01 | 177.11 | -0.72 | 1.000 |
| 20–26-y F - 8–11-y CP | -0.01 | 0.01 | 177.11 | -1.29 | 1.000 |
| 20–26-y F - 14–18-y CP | 0.01 | 0.01 | 177.11 | 1.62 | 1.000 |
| 20–26-y F - 20–26-y CP | 0.01 | 0.00 | 238.15 | 2.36 | 1.000 |
| 20–26-y F - 5–6-y O | 0.00 | 0.01 | 177.11 | 0.16 | 1.000 |
| 20–26-y F - 8–11-y O | -0.00 | 0.01 | 177.11 | -0.50 | 1.000 |
| 20–26-y F - 14–18-y O | 0.04 | 0.01 | 177.11 | 6.64 | **< .001** |
| 20–26-y F - 20–26-y O | 0.05 | 0.00 | 238.15 | 9.62 | **< .001** |
| 5–6-y CP - 8–11-y CP | -0.00 | 0.01 | 177.11 | -0.57 | 1.000 |
| 5–6-y CP - 14–18-y CP | 0.01 | 0.01 | 177.11 | 2.60 | .670 |
| 5–6-y CP - 20–26-y CP | 0.02 | 0.01 | 177.11 | 2.58 | .714 |
| 5–6-y CP - 5–6-y O | 0.01 | 0.00 | 238.15 | 1.27 | 1.000 |
| 5–6-y CP - 8–11-y O | 0.00 | 0.01 | 177.11 | 0.29 | 1.000 |
| 5–6-y CP - 14–18-y O | 0.04 | 0.01 | 177.11 | 8.08 | **< .001** |
| 5–6-y CP - 20–26-y O | 0.05 | 0.01 | 177.11 | 8.31 | **< .001** |
| 8–11-y CP - 14–18-y CP | 0.02 | 0.00 | 238.15 | 4.68 | **< .001** |
| 8–11-y CP - 20–26-y CP | 0.02 | 0.01 | 177.11 | 3.27 | .087 |
| 8–11-y CP - 5–6-y O | 0.01 | 0.01 | 177.11 | 1.58 | 1.000 |
| 8–11-y CP - 8–11-y O | 0.00 | 0.00 | 238.15 | 1.27 | 1.000 |
| 8–11-y CP - 14–18-y O | 0.04 | 0.00 | 238.15 | 12.77 | **< .001** |
| 8–11-y CP - 20–26-y O | 0.05 | 0.01 | 177.11 | 9.37 | **< .001** |
| 14–18-y CP - 20–26-y CP | 0.00 | 0.01 | 177.11 | 0.36 | 1.000 |
| 14–18-y CP - 5–6-y O | -0.01 | 0.01 | 177.11 | -1.59 | 1.000 |
| 14–18-y CP - 8–11-y O | -0.01 | 0.00 | 238.15 | -3.41 | **.050** |
| 14–18-y CP - 14–18-y O | 0.03 | 0.00 | 238.15 | 8.09 | **< .001** |
| 14–18-y CP - 20–26-y O | 0.04 | 0.01 | 177.11 | 6.46 | **< .001** |
| 20–26-y CP - 5–6-y O | -0.01 | 0.01 | 177.11 | -1.71 | 1.000 |
| 20–26-y CP - 8–11-y O | -0.01 | 0.01 | 177.11 | -2.48 | .933 |
| 20–26-y CP - 14–18-y O | 0.03 | 0.01 | 177.11 | 4.66 | **< .001** |
| 20–26-y CP - 20–26-y O | 0.03 | 0.00 | 238.15 | 7.26 | **< .001** |
| 5–6-y O - 8–11-y O | -0.00 | 0.01 | 177.11 | -0.73 | 1.000 |
| 5–6-y O - 14–18-y O | 0.04 | 0.01 | 177.11 | 7.07 | **< .001** |
| 5–6-y O - 20–26-y O | 0.04 | 0.01 | 177.11 | 7.44 | **< .001** |
| 8–11-y O - 14–18-y O | 0.04 | 0.00 | 238.15 | 11.50 | **< .001** |
| 8–11-y O - 20–26-y O | 0.05 | 0.01 | 177.11 | 8.58 | **< .001** |
| 14–18-y O - 20–26-y O | 0.01 | 0.01 | 177.11 | 1.44 | 1.000 |

*Note*. Degrees of freedom were determined using Satterthwaite’s method. *P_adj_*-values are Bonferroni corrected. *SE*= standard error, *df*= degrees of freedom. F = frontal, CP = centro-parietal, O = occipital.

### Supplementary file 1q

*Type III analysis of variance table from a linear mixed-effects model on the effects of age group and slow oscillation topography on slow oscillation density*

| Predictor | Sum of squares | Mean square | *df*_Num_ | *df*_Den_ | *F* | *p* |
| --- | --- | --- | --- | --- | --- | --- |
| Age group | 94.60 | 31.53 | 3 | 96.72 | 64.23 | < .001 |
| Topography | 4.21 | 2.10 | 2 | 240.58 | 4.29 | .015 |
| Age group*topography | 2.55 | 0.43 | 6 | 240.58 | 0.87 | .520 |

*Note*. *df*_Num_ indicates the degrees of freedom numerator. *df*_Den_ indicates the degrees of freedom denominator. Degrees of freedom were determined using Satterthwaite’s method.

### Supplementary file 1r

*Post-hoc comparisons for the effect of age group on slow oscillation density values based on estimated marginal means*

| **A** | |  | |  | |  | |  | |  | |
| --- | --- | --- | --- | --- | --- | --- | --- | --- | --- | --- | --- |
| Contrast | Estimate | | *SE* | | *df* | | *t* | | *p_adj_* | | |
| 5–6-year-olds – 8–11-year-olds | 0.89 | | 0.27 | | 78.34 | | 3.30 | | | | .009 |
| 5–6-year-olds – 14–18-year-olds | -0.47 | | 0.27 | | 78.34 | | -1.75 | | | | .508 |
| 5–6-year-olds – 20–26-year-olds | 0.79 | | 0.31 | | 78.34 | | 2.51 | | | | .085 |
| 8–11-year-olds – 14–18-year-olds | -1.36 | | 0.10 | | 240.58 | | -13.64 | | | | < .001 |
| 8–11-year-olds – 20–26-year-olds | -0.10 | | 0.29 | | 78.34 | | -0.35 | | | | 1.000 |
| 14–18-year-olds – 20–26-year-olds | 1.26 | | 0.29 | | 78.34 | | 4.27 | | | | < .001 |

| **B** | |  | |  |  | |  |  |
| --- | --- | --- | --- | --- | --- | --- | --- | --- |
| Contrast | Estimate | | *SE* | | *df* | *t* | | *p_adj_* |
| F - CP | 0.29 | | 0. 10 | | 241 | 2.93 | | .011 |
| F - O | 0.14 | | 0. 10 | | 241 | 1.40 | | .491 |
| CP -O | -0.15 | | 0. 10 | | 241 | -1.53 | | .382 |

*Note*. Degrees of freedom were determined using Satterthwaite’s method. *P_adj_*-values are Bonferroni corrected. *SE*= standard error, *df*= degrees of freedom.

### Supplementary file 1s

*Type III analysis of variance table from a linear mixed-effects model on the effects of age group and slow oscillation topography on slow oscillation amplitude*

| Predictor | Sum of squares | Mean square | *df*_Num_ | *df*_Den_ | *F* | *p* |
| --- | --- | --- | --- | --- | --- | --- |
| Age group | 320,061.39 | 106,687.13 | 3 | 100.86 | 152.19 | < .001 |
| Topography | 234,095.17 | 117,047.58 | 2 | 243.38 | 166.97 | < .001 |
| Age group*topography | 104,749.27 | 17,458.21 | 6 | 243.38 | 24.91 | < .001 |

*Note*. *df*_Num_ indicates the degrees of freedom numerator. *df*_Den_ indicates the degrees of freedom denominator. Degrees of freedom were determined using Satterthwaite’s method.

### Supplementary file 1t

*Post-hoc comparisons for the slow oscillation amplitude interaction effect based on estimated marginal means*

| Contrast | Estimate | *SE* | *df* | *t* | *p*_adj_ |
| --- | --- | --- | --- | --- | --- |
| 5–6-y F - 8–11-y F | -81.08 | 10.89 | 149.89 | -7.45 | **< .001** |
| 5–6-y F - 14–18-y F | -24.01 | 10.89 | 149.89 | -2.21 | 1.000 |
| 5–6-y F - 20–26-y F | 40.39 | 12.65 | 149.89 | 3.19 | .113 |
| 5–6-y F - 5–6-y CP | 1.44 | 7.64 | 243.38 | 0.19 | 1.000 |
| 5–6-y F - 8–11-y CP | -6.31 | 10.89 | 149.89 | -0.58 | 1.000 |
| 5–6-y F - 14–18-y CP | 60.76 | 10.89 | 149.89 | 5.58 | **< .001** |
| 5–6-y F - 20–26-y CP | 99.33 | 12.65 | 149.89 | 7.85 | **< .001** |
| 5–6-y F - 5–6-y O | -7.38 | 7.64 | 243.38 | -0.97 | 1.000 |
| 5–6-y F - 8–11-y O | -11.98 | 10.89 | 149.89 | -1.10 | 1.000 |
| 5–6-y F - 14–18-y O | 80.61 | 10.89 | 149.89 | 7.41 | **< .001** |
| 5–6-y F - 20–26-y O | 122.38 | 12.65 | 149.89 | 9.67 | **< .001** |
| 8–11-y F - 14–18-y F | 57.06 | 6.52 | 243.38 | 8.75 | **< .001** |
| 8–11-y F - 20–26-y F | 121.47 | 11.89 | 149.89 | 10.22 | **< .001** |
| 8–11-y F - 5–6-y CP | 82.52 | 10.89 | 149.89 | 7.58 | **< .001** |
| 8–11-y F - 8–11-y CP | 74.76 | 6.52 | 243.38 | 11.47 | **< .001** |
| 8–11-y F - 14–18-y CP | 141.83 | 6.52 | 243.38 | 21.76 | **< .001** |
| 8–11-y F - 20–26-y CP | 180.40 | 11.89 | 149.89 | 15.17 | **< .001** |
| 8–11-y F - 5–6-y O | 73.70 | 10.89 | 149.89 | 6.77 | **< .001** |
| 8–11-y F - 8–11-y O | 69.09 | 6.52 | 243.38 | 10.60 | **< .001** |
| 8–11-y F - 14–18-y O | 161.69 | 6.52 | 243.38 | 24.81 | **< .001** |
| 8–11-y F - 20–26-y O | 203.46 | 11.89 | 149.89 | 17.11 | **< .001** |
| 14–18-y F - 20–26-y F | 64.41 | 11.89 | 149.89 | 5.42 | **< .001** |
| 14–18-y F - 5–6-y CP | 25.46 | 10.89 | 149.89 | 2.34 | 1.000 |
| 14–18-y F - 8–11-y CP | 17.70 | 6.52 | 243.38 | 2.72 | .468 |
| 14–18-y F - 14–18-y CP | 84.77 | 6.52 | 243.38 | 13.01 | **< .001** |
| 14–18-y F - 20–26-y CP | 123.34 | 11.89 | 149.89 | 10.37 | **< .001** |
| 14–18-y F - 5–6-y O | 16.64 | 10.89 | 149.89 | 1.53 | 1.000 |
| 14–18-y F - 8–11-y O | 12.03 | 6.52 | 243.38 | 1.85 | 1.000 |
| 14–18-y F - 14–18-y O | 104.63 | 6.52 | 243.38 | 16.05 | **< .001** |
| 14–18-y F - 20–26-y O | 146.40 | 11.89 | 149.89 | 12.31 | **< .001** |
| 20–26-y F - 5–6-y CP | -38.95 | 12.65 | 149.89 | -3.08 | .163 |
| 20–26-y F - 8–11-y CP | -46.70 | 11.89 | 149.89 | -3.93 | **.009** |
| 20–26-y F - 14–18-y CP | 20.36 | 11.89 | 149.89 | 1.71 | 1.000 |
| 20–26-y F - 20–26-y CP | 58.94 | 8.83 | 243.38 | 6.68 | **< .001** |
| 20–26-y F - 5–6-y O | -47.77 | 12.65 | 149.89 | -3.78 | **.015** |
| 20–26-y F - 8–11-y O | -52.37 | 11.89 | 149.89 | -4.41 | **.001** |
| 20–26-y F - 14–18-y O | 40.22 | 11.89 | 149.89 | 3.38 | .060 |
| 20–26-y F - 20–26-y O | 81.99 | 8.83 | 243.38 | 9.29 | **< .001** |
| 5–6-y CP - 8–11-y CP | -7.76 | 10.89 | 149.89 | -0.71 | 1.00 |
| 5–6-y CP - 14–18-y CP | 59.31 | 10.89 | 149.89 | 5.45 | **< .001** |
| 5–6-y CP - 20–26-y CP | 97.88 | 12.65 | 149.89 | 7.74 | **< .001** |
| 5–6-y CP - 5–6-y O | -8.82 | 7.64 | 243.38 | -1.15 | 1.000 |
| 5–6-y CP - 8–11-y O | -13.43 | 10.89 | 149.89 | -1.23 | 1.000 |
| 5–6-y CP - 14–18-y O | 79.17 | 10.89 | 149.89 | 7.27 | **< .001** |
| 5–6-y CP - 20–26-y O | 120.94 | 12.65 | 149.89 | 9.56 | **< .001** |
| 8–11-y CP - 14–18-y CP | 67.07 | 6.52 | 243.38 | 10.29 | **< .001** |
| 8–11-y CP - 20–26-y CP | 105.64 | 11.89 | 149.89 | 8.89 | **< .001** |
| 8–11-y CP - 5–6-y O | -1.06 | 10.89 | 149.89 | -0.10 | 1.000 |
| 8–11-y CP - 8–11-y O | -5.67 | 6.52 | 243.38 | -0.87 | 1.000 |
| 8–11-y CP - 14–18-y O | 86.93 | 6.52 | 243.38 | 13.34 | **< .001** |
| 8–11-y CP - 20–26-y O | 128.70 | 11.89 | 149.89 | 10.82 | **< .001** |
| 14–18-y CP - 20–26-y CP | 38.57 | 11.89 | 149.89 | 3.24 | .096 |
| 14–18-y CP - 5–6-y O | -68.13 | 10.89 | 149.89 | -6.26 | **< .001** |
| 14–18-y CP - 8–11-y O | -72.74 | 6.52 | 243.38 | -11.16 | **< .001** |
| 14–18-y CP - 14–18-y O | 19.86 | 6.52 | 243.38 | 3.05 | .170 |
| 14–18-y CP - 20–26-y O | 61.63 | 11.89 | 149.89 | 5.18 | **< .001** |
| 20–26-y CP - 5–6-y O | -106.71 | 12.65 | 149.89 | -8.43 | **< .001** |
| 20–26-y CP - 8–11-y O | -111.31 | 11.89 | 149.89 | -9.36 | **< .001** |
| 20–26-y CP - 14–18-y O | -18.71 | 11.89 | 149.89 | -1.57 | 1.000 |
| 20–26-y CP - 20–26-y O | 23.06 | 8.83 | 243.38 | 2.61 | .630 |
| 5–6-y O - 8–11-y O | -4.60 | 10.89 | 149.89 | -0.42 | 1.000 |
| 5–6-y O - 14–18-y O | 87.99 | 10.89 | 149.89 | 8.08 | **< .001** |
| 5–6-y O - 20–26-y O | 129.76 | 12.65 | 149.89 | 10.26 | **< .001** |
| 8–11-y O - 14–18-y O | 92.60 | 6.52 | 243.38 | 14.21 | **< .001** |
| 8–11-y O - 20–26-y O | 134.37 | 11.89 | 149.89 | 11.30 | **< .001** |
| 14–18-y O - 20–26-y O | 41.77 | 11.89 | 149.89 | 3.51 | **.039** |

*Note*. Degrees of freedom were determined using Satterthwaite’s method. *P_adj_*-values are Bonferroni corrected. *SE*= standard error, *df*= degrees of freedom. F = frontal, CP = centro-parietal, O = occipital.

# Association between sleep spindle and slow oscillation maturity with modulation strength

### Supplementary file 1u

*Results of the generalized linear mixed-effects model on the association between the fast sleep spindle maturity component and frontal slow oscillation-development-specific fast spindle modulation strength*

| Predictor | Estimate | Standard error | *t* | *p* |
| --- | --- | --- | --- | --- |
| (Intercept) | -1.78 | 0.07 | -26.78 | < .001 |
| Fast sleep spindle maturity component | 0.38 | 0.06 | 6.90 | < .001 |
| Slow oscillation maturity score | 0.09 | 0.07 | 1.44 | .150 |

*Note*. Model was fit with gamma errors and a log link function.

### Supplementary file 1v

*Results of the generalized linear mixed-effects model on the association between the fast sleep spindle maturity component and (A) centro-parietal and (B) frontal slow oscillation-development-specific fast spindle modulation strength while also controlling for age*

| **A** |  |  |  |  |
| --- | --- | --- | --- | --- |
| Predictor | Estimate | Standard error | *t* | *p* |
| (Intercept) | -1.81 | 0.08 | -23.82 | < .001 |
| Fast sleep spindle maturity component | 0.25 | 0.08 | 3.00 | .003 |
| Slow oscillation maturity score | 0.002 | 0.07 | 0.03 | .977 |
| Age (in months) | 0.32 | 0.09 | 3.76 | < .001 |
| **B** |  |  |  |  |
| Predictor | Estimate | Standard error | *t* | *p* |
| (Intercept) | -1.79 | 0.07 | -27.28 | < .001 |
| Fast sleep spindle maturity component | 0.25 | 0.08 | 3.11 | .002 |
| Slow oscillation maturity score | 0.04 | 0.07 | 0.64 | .526 |
| Age (in months) | 0.18 | 0.08 | 2.23 | .026 |

*Note*. Model was fit with gamma errors and a log link function.

# Sleep architecture

### Supplementary file 1w

Sleep architecture

|  | 5–6-year-olds | | | | 8–11-year-olds | | | | 14–18-year-olds | | | | 20–26-year-olds | | | | |
| --- | --- | --- | --- | --- | --- | --- | --- | --- | --- | --- | --- | --- | --- | --- | --- | --- | --- |
|  | | Mean | Median | Percentile [25%; 75%] | | Mean | Median | Percentile [25%; 75%] | | Mean | Median | Percentile [25%; 75%] | | Mean | Median | Percentile [25%; 75%] | |
| TST (min) | | 605.52 | 610.00 | [593.88; 629.12] | | 560.44 | 565.50 | [540.50; 590.00] | | 474.70 | 486.50 | [440.50; 505.00] | | 445.42 | 451.50 | [424.25 ; 477.25 ] | |
| N1(%) | | 5.95 | 5.19 | [3.96;7.33] | | 2.49 | 2.21 | [1.32; 3.36] | | 8.69 | 7.60 | [5.26; 11.17] | | 11.63 | 11.10 | [8.62;14.76 ] | |
| N2(%) | | 35.88 | 35.40 | [34.06; 39.20] | | 43.62 | 42.87 | [39.15; 49.78] | | 40.70 | 39.85 | [37.63; 43.27] | | 49.80 | 47.74 | [45.81 ; 52.49 ] | |
| N3(%) | | 24.73 | 23.82 | [21.21; 26.96] | | 26.61 | 26.46 | [21.78; 32.57] | | 25.38 | 23.65 | [20.80; 28.09] | | 18.64 | 19.26 | [15.16; 23.95 ] | |
| R(%) | | 33.44 | 33.67 | [30.27; 34.80] | | 27.27 | 25.86 | [22.01; 31.08] | | 25.24 | 25.42 | [21.21; 29.64] | | 19.93 | 20.32 | [15.54 ; 23.95] | |
| WASO (min) | | 10.33 | 5.75 | [4.00; 9.12] | | 10.06 | 6.50 | [2.50; 13.00] | | 10.55 | 9.00 | [4.00; 16.00] | | 18.31 | 7.25 | [5.12 ; 11.12] | |
| NREM (%) | | 60.61 | 61.15 | [56.48; 64.50] | | 70.23 | 71.48 | [67.03; 75.12] | | 66.07 | 65.49 | [62.54; 70.60] | | 68.44 | 68.73 | [65.83; 71.38 ] | |
| *Note*. TST=Total sleep time, WASO=Wake after sleep onset, R=rapid-eye movement sleep, NREM=sum of N2 and N3 sleep. | | | | | | | | | | | | | | | | |  |

# Co-occurrence of sleep spindles and slow oscillations and vice versa

### Supplementary file 1x

*Type III analysis of variance table from a linear mixed-effects model on the effects of age group and slow oscillation topography on the co-occurrence of slow frontal sleep spindles with slow oscillations*

| Predictor | Sum of squares | Mean square | *df*_Num_ | *df*_Den_ | *F* | *p* |
| --- | --- | --- | --- | --- | --- | --- |
| Age group | 147.97 | 49.32 | 3 | 92.33 | 8.95 | .<.001 |
| Topography | 2,077.16 | 1,083.58 | 2 | 237.14 | 188.55 | < .001 |
| Age group*topography | 679.67 | 113.28 | 6 | 237.14 | 20.57 | < .001 |

*Note*. Model formula: measurement ~ age group * topography + (1 | ID), F_SPCoup_Data, TRUE, lmerControl (optimizer = “nloptwrap”, optCtrl = list(algorithm = “NLOPT_LN_BOBYQA”)), na.exclude. *df*_Num_ indicates the degrees of freedom numerator. *df*_Den_ indicates the degrees of freedom denominator. Degrees of freedom were determined using Satterthwaite’s method.

### Supplementary file 1y

*Type III analysis of variance table from a linear mixed-effects model on the effects of age group and slow oscillation topography on the co-occurrence of development-specific fast centro-parietal sleep spindles with slow oscillations*

| Predictor | Sum of squares | Mean square | *df*_Num_ | *df*_Den_ | F | p |
| --- | --- | --- | --- | --- | --- | --- |
| Age group | 35.93 | 11.98 | 3 | 95.40 | 3.27 | .025 |
| Topography | 1,545.45 | 772.72 | 2 | 238.46 | 210.67 | < .001 |
| Age group*topography | 368.33 | 61.39 | 6 | 238.46 | 16.74 | < .001 |

*Note*. Model formula: measurement ~ age group * topography + (1 | ID), CP_SPCoup_Data, TRUE, lmerControl(optimizer = “nloptwrap”, optCtrl = list(algorithm = “NLOPT_LN_BOBYQA”)), na.exclude. *df*_Num_ indicates the degrees of freedom numerator. *df*_Den_ indicates the degrees of freedom denominator. Degrees of freedom were determined using Satterthwaite’s method.

### Supplementary file 1z

*Type III analysis of variance table from a linear mixed-effects model on the effects of age group and slow oscillation topography on the co-occurrence of slow oscillations with slow frontal sleep spindles*

| Predictor | Sum of squares | Mean square | *df*_Num_ | *df*_Den_ | *F* | *p* |
| --- | --- | --- | --- | --- | --- | --- |
| Age group | 2.42 | 0. 81 | 3 | 94.14 | 1.21 | .312 |
| Topography | 94.65 | 47.33 | 2 | 239.67 | 70.63 | < .001 |
| Age group*topography | 21.84 | 3.51 | 6 | 239.67 | 5.43 | < .001 |

*Note*. Model formula: lmer, measurement ~ age group * topography + (1 | ID), SOCoup_Data_FSP, TRUE, lmerControl(optimizer = “nloptwrap”, optCtrl = list(algorithm = “NLOPT_LN_BOBYQA”)), na.exclude. *df*_Num_ indicates the degrees of freedom numerator. *df*_Den_ indicates the degrees of freedom denominator. Degrees of freedom were determined using Satterthwaite’s method.

### Supplementary file 1aa

*Type III analysis of variance table from a linear mixed-effects model on the effects of age group and slow oscillation topography on the co-occurrence of slow oscillations with development-specific fast centro-parietal sleep spindles*

| Predictor | Sum of squares | Mean square | *df*_Num_ | *df*_Den_ | *F* | *p* |
| --- | --- | --- | --- | --- | --- | --- |
| Age group | 7.38 | 2.46 | 3 | 95.02 | 4.03 | . 010 |
| Topography | 101.68 | 50.84 | 2 | 239.89 | 83.25 | < .001 |
| Age group*topography | 16.24 | 2.71 | 6 | 239.89 | 4.42 | < .001 |

*Note*. Model formula: lmer, measurement ~ age group * topography + (1 | ID), SOCoup_Data_CPSP, TRUE, lmerControl(optimizer = “nloptwrap”, optCtrl = list(algorithm = “NLOPT_LN_BOBYQA”)), na.exclude. *df*_Num_ indicates the degrees of freedom numerator. *df*_Den_ indicates the degrees of freedom denominator. Degrees of freedom were determined using Satterthwaite’s method.

### Supplementary file 1ab

*Type III analysis of variance table from a linear mixed-effects model on the effects of age group and slow oscillation topography on the co-occurrence of adult-like fast centro-parietal sleep spindles with slow oscillations*

| Predictor | Sum of squares | Mean square | *df*_Num_ | *df*_Den_ | *F* | *p* |
| --- | --- | --- | --- | --- | --- | --- |
| Age group | 304.85 | 101.62 | 3 | 96.62 | 16.08 | < .001 |
| Topography | 2,284.98 | 1,142.49 | 2 | 239.68 | 180.76 | < .001 |
| Age group*topography | 531.23 | 88.54 | 6 | 239.68 | 14.01 | < .001 |

*Note*. Model formula: measurement ~ age group * topography + (1 | ID), SPCoup_Data_adult, TRUE, lmerControl (optimizer = “nloptwrap”, optCtrl = list(algorithm = “NLOPT_LN_BOBYQA”)), na.exclude. *df*_Num_ indicates the degrees of freedom numerator. *df*_Den_ indicates the degrees of freedom denominator. Degrees of freedom were determined using Satterthwaite’s method.

### Supplementary file 1ac

*Type III analysis of variance table from a linear mixed-effects model on the effects of age group and slow oscillation topography on the co-occurrence of slow oscillations with adult-like fast centro-parietal sleep spindles*

| Predictor | Sum of squares | Mean square | *df*_Num_ | *df*_Den_ | *F* | *p* |
| --- | --- | --- | --- | --- | --- | --- |
| Age group | 22.49 | 7.50 | 3 | 94.89 | 11.66 | < .001 |
| Topography | 98.67 | 49.48 | 2 | 239.79 | 76.98 | < .001 |
| Age group*topography | 17.06 | 2.84 | 6 | 239.79 | 4.43 | < .001 |

*Note*. Model formula: measurement ~ age group * topography + (1 | ID), SOCoup_Data_adult, TRUE, lmerControl (optimizer = “nloptwrap”, optCtrl = list(algorithm = “NLOPT_LN_BOBYQA”)), na.exclude. *df*=degrees of freedom. *df*_Num_ indicates the degrees of freedom numerator. *df*_Den_ indicates the degrees of freedom denominator. Degrees of freedom were determined using Satterthwaite’s method.
